# Clinical predictors of protracted length of stay in Ontario Complex Continuing Care hospitals

**DOI:** 10.1186/s12913-019-4024-2

**Published:** 2019-04-05

**Authors:** Luke A. Turcotte, Chris M. Perlman, Brant E. Fries, John P. Hirdes

**Affiliations:** 1 School of Public Health and Health Systems, University of Waterloo, 200 University Avenue West, Waterloo, ON N2L 3G1 Canada; 2 Geriatrics Center, Department of Internal Medicine and School of Public Health, University of Michigan, Ann Arbor, USA

**Keywords:** Patient flow, Length of stay, Post-acute care, Discharge planning

## Abstract

**Background:**

Post-acute care hospitals are often subject to patient flow pressures because of their intermediary position along the continuum of care between acute care hospitals and community care or residential long-term care settings. The purpose of this study was to identify patient attributes associated with a prolonged length of stay in Complex Continuing Care hospitals.

**Methods:**

Using information collected using the interRAI Resident Assessment Instrument Minimum Data Set 2.0 (MDS 2.0), a sample of 91,113 episodes of care for patients admitted to Complex Continuing Care hospitals between March 31, 2001 and March 31, 2013 was established. All patients in the sample were either discharged to a residential long-term care facility (e.g., nursing home) or to the community. Long-stay patients for each discharge destination were identified based on a length of stay in the 95th percentile. A series of multivariate logistic regression models predicting long-stay patient status for each discharge destination pathway were fit to characterize the association between demographic factors, residential history, health severity measures, and service utilization on prolonged length of stay in post-acute care.

**Results:**

Risk factors for prolonged length of stay in the adjusted models included functional and cognitive impairment, greater pressure ulcer risk, paralysis, antibiotic resistant and HIV infection need for a feeding tube, dialysis, tracheostomy, ventilator or a respirator, and psychological therapy. Protective factors included advanced age, medical instability, a greater number of recent hospital and emergency department visits, cancer diagnosis, pneumonia, unsteady gait, a desire to return to the community, and a support person who is positive towards discharge. Aggressive behaviour was only a risk factor for patients discharged to residential long-term care facilities. Cancer diagnosis, antibiotic resistant and HIV infection, and pneumonia were only significant factors for patients discharged to the community.

**Conclusions:**

This study identified several patient attributes and process of care variables that are predictors of prolonged length of stay in post-acute care hospitals. This is valuable information for care planners and health system administrators working to improve patient flow in Complex Continuing Care and other post-acute care settings such as skilled nursing and inpatient rehabilitation facilities.

## Introduction

The Canadian health care system comprises a series of primary, secondary, and tertiary service settings organized along a “continuum of care.” Individuals with chronic and complex health conditions may access several of these services settings during an episode of care [1]. With the aim of reducing costs and ensuring timely access to care for all system users, patients are transitioned to the most appropriate service setting relative to their needs in time. Seamless boundaries between contiguous service settings are the aim; however, the reality of an integrated health system has not yet been realized [2].

In Ontario, Canada, Complex Continuing Care (CCC) hospitals (akin to skilled nursing facilities in other health systems) provide nursing and rehabilitation care to patients with complex medical needs after hospitalization in an acute-care hospital [3]. As an intermediary setting along the continuum of care, CCC hospitals often discharge patients to community care or residential long-term care (LTC) settings such as nursing homes; however, more than a third of patients die in these facilities [4]. The median length of stay for patients in CCC facilities ranges between 28 and 31 days by age group [5]. Although episodes of care are short for most CCC patients, more than 40% stay longer than 90 days [6].

Rates of delayed discharge in Ontario CCC facilities are high, with more than 20% of beds occupied by patients designated as requiring Alternate Level of Care (ALC) [7]. ALC is used in Ontario and other Canadian provinces to identify patients that are receiving care in a setting where the intensity and type of care that is offered is no longer appropriate for their current needs [8]. Though not all CCC patients are eligible to transition to the community or an LTC facility, identifying the subgroup of patients that require support to transition is necessary, as these patients are at greater risk of delayed discharge [9].

Previous studies have contrasted short-stay and long-stay patients in post-acute care settings. Long-stay patients, usually defined as those with a length of stay of 90 days or more, are typically older patients [10, 11] with greater physical [11–13] or cognitive impairment [12, 13] (including dementia [10, 13]), a mood disorder [13], or a recent fall [9]. Factors such as clinical instability and delirium may prolong the hospital length of stay [14]. Discharge destination influences length of stay [15]. In part, this is because admission to a residential long-term care is often subject to delays from family deliberations, accommodation availability and funding arrangements [16].

This study examined patients in Ontario CCC facilities with a length of stay in the 95th percentile to identify clinical characteristics at admission that are predictors of protracted length of stay. This extreme definition of long-stay status is intended to identify individuals for whom transition to the community or LTC is unlikely without substantial gains in function or extensive discharge planning. By targeting patients with the longest lengths of stay, this study focused on those likely to consume a disproportional amount of the overall hospital bed days and provides information for policy decisions to build system capacity.

## Methods

### Sample

interRAI Resident Assessment Instrument Minimum Data Set 2.0 (MDS 2.0) assessments from the Continuing Care Reporting System (CCRS) data repository were used as the data source for this retrospective study. The CCRS is maintained by the Canadian Institute for Health Information and includes MDS 2.0 assessments from LTC and CCC facilities in nine Canadian provinces and territories [17]. The MDS 2.0 is a comprehensive clinical assessment used for patient care planning, case-mix funding, and health system performance measurement [18–21].

The sample consisted of patients assessed in CCC facilities between March 31, 2001 and March 31, 2013 that were discharged to the community or an LTC facility. Excluding patients that died in CCC or were discharged to other care settings, such as acute care hospitals, ensured that all patients in the sample were eligible to transition to a less resource-intensive care setting. Given that CCC is a transitional care setting, patients may be admitted to higher or lower levels of care for short periods of time before returning to a CCC facility for the remainder of their care. Therefore, the level of analysis was among CCC episodes of care during the study period, not individuals. An episode of care was defined as the period in which a patient receives care in a CCC facility without a temporary discharge of more than 14 days. Patients that were temporarily discharged for more than 14 days were assigned a new episode of care upon return to the CCC facility. A final analytic sample of 91,113 episodes of care, representing 82,174 individuals, was used to the complete the analysis for this study. Among all individuals admitted to CCC facilities during the sample time frame, 7.6% contributed two episodes of care to the final analytic sample, with an additional 1.3% contributing three or more episodes of care. For individuals contributing two or more episodes of care, a median of 350 days (IQR 131–858 days) separated the episodes of care.

Given that there are differences in the discharge planning processes for community and LTC-bound patients, the total sample was divided into two discharge destination sub-samples. The community discharge sub-sample included patients discharged home with or without home care services or patients discharged to a community-based facility providing board and care, such as a retirement home. Patients in this sub-sample accounted for 58,816 (64.6%) episodes of care. The LTC dishcharge sub-sample included patients discharged to a residential care facility that offers 24-hour nursing care, representing 32,297 (35.4%) episodes of care.

### Dependent and independent variables

Long-stay patient status, defined as an episode of care length of stay within the 95th percentile, was used as the binary dependent variable in this study. Length of stay was calculated as the difference between the episode of care admission and discharge dates. For both discharge destination sub-samples, the 95th percentile long-stay threshold was calculated among only the patients belonging to the particular sub-sample, meaning that the definition of the long-stay dependent variable differs between the overall sample, community discharge and LTC discharge sub-samples.

Independent variables to predict long-stay patient status were selected from the MDS 2.0 admission assessment based on prior literature and consultation through expert clinical input. These independent variables included sociodemographic characteristics: age and marital status; residential history in the five years preceding the start of the episode of care, desire to return to the community and availability of informal support, number of emergency department visits and hospitalizations in the preceding 90 days, diseases and other health conditions available as checklist items on the assessment, and provision of treatments and therapies. Also, embedded within the MDS 2.0 assessment are a series of outcome measures and summary scales. The following outcome measures and summary scales were also used as independent variables in this analysis: ADL Hierarchy Scale [22], Cognitive Performance Scale (CPS) [23], Depression Rating Scale [24], Changes in Health, End-Stage Disease, Signs and Symptoms Scale (CHESS) [25], Aggressive Behaviour Scale [26], Pressure Ulcer Risk Scale [27], Pain Scale [28] and the Index of Social Engagement [29, 30]. The validity and reliability of items and scales embedded within the MDS 2.0 assessment have been studied at depth [31] and the quality of the data that is collected using the assessment is strong [32].

### Statistical analyses

Descriptive statistics were calculated for pertinent MDS 2.0 clinical variables, outcome measures, and scales to compare regular and long-stay patients with the samples. Chi-square (*χ*^2^) tests were used to determine the statistical significance of differences in the frequency response between length of stay groups for binary and categorical variables of interest. To account for multiple comparisons, a Bonferroni correction was made. This resulted in an adjusted alpha of 0.05/126 = 0.0004. The MDS 2.0 admission assessment for each episode of care included in the study sample was used to calculate the sample characteristics for both lengths of stay groups. Subsequently, a series of multivariate logistic regression models were created to predict length of stay in the 95th percentile within the overall sample and the discharge destination sub-samples. Model candidate variables were selected for inclusion based on results from descriptive statistics and bivariate logistic regression analyses. All analyses were completed using SAS 9.4 (SAS Institute, Cary NC).

## Results

### Descriptive statistics

The median episode length among patients in the overall sample was 44 days (IQR 26–83 days). Patients in the community discharge sub-sample had a shorter median episode length (38 days, IQR 23–64 days) compared to patients in the LTC discharge sub-sample (63 days, IQR 33–127 days). In the overall sample, patients whose episode of care was 235 days or greater (representing the 95th percentile) were designated as long-stay patients. The 95th percentile length of stay threshold for patients in the community and LTC discharge sub-samples was 154 and 362 days, respectively.

Table 1 presents sociodemographic characteristics for regular and long-stay CCC patients by discharge destination. In the overall sample, long-stay patients were younger and more likely to be male, more likely to speak a foreign language and less likely to have lived alone before entering the CCC facility. More than three-quarters of the regular-stay patients in the overall sample had a desire to return to the community compared to only 58% of the long-stay patients. Fewer long-stay patients had a support person that was positive towards discharge.

**Table 1:** Patient sociodemographics, residential history, recent health service use, health conditions, and discharge potential by length of stay group and discharge destination

	Overall (*n* = 91,113)		Community Discharge (*n* = 58,816)		LTC Discharge (*n* = 32,297)	
Length of Stay Group	Regular-stay	Long-stay (235+ days)	Regular-stay	Long-say (154+ days)	Regular-stay	Long-stay (362+ days)
Age Group						
0–64	13%	22%	15%	30%	6%	21%
65–74	16%	18%	18%	22%	12%	18%
75–84	38%	35%	38%	30%	39%	36%
85–94	30%	23%	26%	17%	38%	23%
95+	3%	3%	2%	1%	4%	3%
Female	62%	56%	62%	54%	63%	56%
Married	37%*	36%*	40%*	40%*	31%*	35%*
Speaks primarily foreign language	8%	10%	8%	12%	8%	11%
Desire to return to community	82%	58%	91%	73%	66%	49%
Support person positive towards discharge	79%	53%	87%	69%	62%	45%
Lived alone prior to entry	31%	27%	30%	26%	32%	27%
Residential History						
LTC facility	8%*	7%*	5%*	5%*	12%	7%
Psychiatric care facility	1%	2%	1%	1%	1%	3%
Post-acute care facility	33%	35%	32%*	33%*	35%*	38%*
Emergency Department Visits						
1 visit in last 90 days	56%	52%	57%	54%	53%	50%
2+ visits in last 90 days	22%	15%	21%	15%	22%	14%
Hospital Admissions						
1 admission in last 90 days	39%	34%	38%	34%	42%	32%
2+ admissions in last 90 days	8%	5%	8%	5%	9%	5%
Diseases						
Alzheimer’s disease or a related dementia	24%	33%	16%	19%	39%*	39%*
Aphasia	4%	12%	3%	10%	6%	17%
Cancer	15%	12%	15%	13%	14%	10%
Emphysema/COPD	16%	13%	17%	12%	16%*	14%*
Hemiplegia/Hemiparesis	8%	16%	7%	20%	10%	17%
Hip fracture	15%	12%	16%	11%	14%*	11%*
Multiple sclerosis	1%	2%	1%	3%	1%	3%
Quadriplegia	0%	2%	1%*	3%*	0%	2%
Schizophrenia	1%	2%	1%*	2%*	2%	3%
Stroke	20%	31%	18%	31%	25%	32%
Traumatic brain injury	1%	4%	1%	4%	1%	5%
Infections						
Antibiotic resistant infection	6%	9%	6%	11%	6%	9%
HIV infection	0.2%	0.6%	0.2%	1.0%	0.1%	0.4%
Pneumonia	7%*	6%*	7%*	6%*	6%*	5%*
Urinary tract infection	19%*	17%*	18%*	16%*	22%	18%
Conditions						
Bowel or bladder incontinence	45%	67%	36%	57%	61%	69%
Hallucinations or delusions	6%	9%	4%	5%	9%*	10%*
Unsteady gait	52%	39%	52%	38%	51%	40%
Treatments & Therapies						
Community skills training	48%	32%	57%	45%	31%	24%
Dialysis	2%*	2%*	2%	3%	1%*	2%*
Feeding tube	3%	9%	2%	9%	3%	11%
Psychological therapy	13%	20%	14%	19%	11%	21%
Tracheostomy, ventilator or respirator	1%	3%	1%	3%	1%	2%
Trunk, limb or chair restraint	12%	20%	7%	15%	20%	22%

All length of stay group-based chi-square statistics are significant to *p* < 0.0004 unless denoted by a *

Among patients in the overall sample, a greater percentage of long-stay patients had a diagnosis of Alzheimer’s disease and related dementias, aphasia, hemiplegia/hemiparesis, multiple sclerosis, quadriplegia, schizophrenia, stroke and traumatic brain injury. These trends did not differ when examining community or LTC discharges independently, except for LTC discharges where an equal percentage of regular and long-stay patients had an Alzheimer’s disease and related dementias diagnosis. Additionally, there was no difference between groups in the percentage of patients with quadriplegia and schizophrenia that were discharged to the community. Fewer long-stay patients had a diagnosis of cancer, emphysema/COPD, and hip fracture. This was also true among patients discharged to the community; however, among there was no difference in the percentage of patients with emphysema/COPD and hip fracture among the patients discharged to LTC. Significantly more long-stay patients in all samples were admitted with antibiotic-resistant infections and HIV. Fewer long-stay patients were admitted with a urinary tract infection among the patients discharged to the community. Long-stay patients were more likely to be admitted with occasional or worse bladder or bowel incontinence, but this difference was greatest among patients in the community discharge sub-sample(Table 1).

Group differences in the percentage of regular and long-stay patients receiving select treatments and therapies were also noted. Long-stay patients were more likely to recieve nutrition through a feeding tube, require tracheostomy, ventilator or respirator, and recieve psychological therapy, but less likely to receive community skills training. Lastly, a greater percentage of long-stay patients were restrained using a trunk, limb or chair restraint (Table 1).

Table 2 presents clinical outcome measures for regular and long-stay patients by discharge destination. Long-stay patients were more impaired in ADL self-performance and cognition, and demonstrated a greater frequency and diversity of aggressive behaviours. Long-stay patients were also more clinically stable than regular-stay patients, as determined by CHESS. Except for patients discharged to LTC, the distribution of Index of Social Engagement scores indicates that long-stay patients were less socially engaged than regular-stay patients (Table 2).

**Table 2:** Clinical measures and scales by length of stay group and discharge destination

	Overall (*n* = 91,113)		Community Discharge (*n* = 58,816)		LTC Discharge (*n* = 32,297)	
Length of Stay Group	Regular-stay	Long-stay (235+ days)	Regular-stay	Long-stay (164+ days)	Regular-stay	Long-stay (362+ days)
ADL Hierarchy Scale						
0	8%	4%	10%	4%	5%	3%
1–2	32%	17%	37%	19%	23%	15%
3–4	28%	31%	27%	34%	31%	30%
5–6	32%	48%	26%	43%	42%	51%
Cognitive Performance Scale						
0	31%	35%	39%	37%	15%	31%
1–2	35%	29%	37%	33%	31%	27%
3–4	24%	31%	18%	26%	36%	31%
5–6	10%	28%	6%	12%	18%	28%
Aggressive Behaviour Scale						
0	79%	67%	84%	75%	70%	60%
1–4	17%	25%	14%	20%	24%	28%
5+	3%	8%	2%	5%	6%	12%
CHESS[1]						
0	10%	27%	21%	27%	18%	27%
1–2	55%	49%	57%	51%	50%	47%
3+	26%	25%	22%	22%	32%	26%
Index of Social Engagement						
0–1	26%	37%	19%	28%	38%*	43%*
2–4	44%	43%	46%	46%	42%*	39%*
5–6	30%	20%	35%	25%	21%*	18%*

All length of stay group-based chi-square statistics are significant to *p <* 0.0004 unless denoted by a *

[1] Changes in Health, End-Stage Disease, Signs, and Symptoms Scale

### Multivariable models

The multivariable logistic regression model predicting the binary dependent variable of long-stay designation is presented in Table 3. Risk factors for long-stay designation included increasing levels of functional impairment (ADL Hierarchy Scale), cognitive impairment (CPS), and pressure ulcer risk, hemiplegia, quadriplegia, traumatic brain injury, aphasia, antibiotic resistant infection, and HIV infection. In addition to clinical status, need for a feeding tube, dialysis, psychological therapy, and tracheostomy, ventilator or respirator were predictive of long-stay designation. Protective factors included female sex, older age, higher levels of clinical instability (CHESS), increasing number of recent hospital stays and emergency department visits, previous nursing home admission, cancer diagnosis, pneumonia, and unsteady gait. Patients that desired to return to the community or had a support person who was positive towards discharge also had lower odds of long-stay designation.

**Table 3:** Multivariable logistic regression model predicting long-stay designation by discharge destination

		Overall		Community Discharge		LTC Discharge	
		Adjusted Odds Ratio (95% CI)	*P*-Value	Adjusted Odds Ratio (95% CI)	*P*-Value	Adjusted Odds Ratio (95% CI)	*P*-Value
Demographics							
Female (ref = male)		0.90 (0.85–0.96)	< 0.01	0.90 (0.83–0.97)	0.01	1.00 (0.90–1.12)	0.96
Age (ref = 0–64)	65–74	0.68 (0.61–0.75)	<.0001	0.69 (0.62–0.77)	<.0001	0.52 (0.44–0.62)	<.0001
	75–84	0.60 (0.55–0.66)	<.0001	0.51 (0.46–0.57)	<.0001	0.38 (0.33–0.44)	<.0001
	85–94	0.52 (0.47–0.58)	<.0001	0.43 (0.38–0.49)	<.0001	0.30 (0.26–0.36)	<.0001
	95+	0.56 (0.46–0.69)	<.0001	0.29 (0.20–0.41)	<.0001	0.35 (0.25–0.49)	<.0001
Clinical Scales							
ADL Hierarchy Scale (ref = 0)	1–2	1.27 (1.08–1.50)	< 0.01	1.59 (1.30–1.94)	<.0001	1.36 (0.99–1.87)	0.06
	3–4	2.11 (1.78–2.49)	<.0001	3.01 (2.46–3.68)	<.0001	1.76 (1.28–2.42)	0.0005
	5–6	2.09 (1.76–2.48)	<.0001	2.73 (2.22–3.36)	<.0001	1.87 (1.35–2.59)	0.0002
Cognitive performance scale (ref = 0)	1–2	1.33 (1.21–1.46)	<.0001	1.08 (0.98–1.19)	0.12	0.94 (0.80–1.12)	0.51
	3+	1.60 (1.46–1.75)	<.0001	1.23 (1.11–1.37)	0.0001	0.80 (0.67–0.94)	0.008
CHESS [1] (ref = 0)	1–2	0.72 (0.66–0.77)	<.0001	0.74 (0.67–0.81)	<.0001	0.72 (0.63–0.82)	<.0001
	3+	0.62 (0.57–0.69)	<.0001	0.65 (0.57–0.74)	<.0001	0.62 (0.53–0.73)	<.0001
Pressure ulcer risk scale (ref = 0)	1–2	1.06 (0.95–1.17)	0.28	1.21 (1.06–1.38)	0.006	1.08 (0.91–1.29)	0.38
	3	1.33 (1.18–1.50)	<.0001	1.72 (1.47–2.00)	<.0001	1.21 (0.98–1.48)	0.08
	4–5	1.49 (1.31–1.70)	<.0001	2.07 (1.75–2.45)	<.0001	1.28 (1.03–1.59)	0.03
	5+	2.00 (1.56–2.56)	<.0001	2.95 (2.14–4.06)	<.0001	1.54 (1.02–2.32)	0.04
Aggressive behaviour scale (ref = 0)	1–4					1.32 (1.16–1.49)	<.0001
	5+					1.81 (1.50–2.17)	<.0001
Health service utilization							
Hospital stays [2] (ref = 0)	1 stay	0.81 (0.75–0.87)	<.0001	0.80 (0.72–0.88)	<.0001	0.78 (0.69–0.89)	0.0002
	2+ stays	0.66 (0.59–0.73)	<.0001	0.63 (0.55–0.72)	<.0001	0.63 (0.52–0.75)	<.0001
Emergency department visits [2] (ref = 0)	1 visit	0.85 (0.79–0.92)	<.0001	0.95 (0.86–1.04)	0.23	0.77 (0.68–0.87)	<.0001
	2+ visits	0.70 (0.60–0.81)	<.0001	0.77 (0.64–0.93)	0.006	0.65 (0.51–0.83)	0.0006
Previous nursing home admission (ref = no)		0.82 (0.72–0.92)	< 0.01			0.63 (0.52–0.76)	<.0001
Health conditions							
Hemiplegia (ref = not present)		1.40 (1.27–1.54)	<.0001	2.27 (2.04–2.53)	<.0001	1.22 (1.05–1.43)	0.01
Quadriplegia (ref = not present)		1.38 (1.07–1.79)	0.01			2.79 (1.73–4.49)	<.0001
Traumatic brain injury (ref = not present)		1.67 (1.38–2.02)	<.0001	1.92 (1.52–2.42)	<.0001	1.82 (1.36–2.44)	<.0001
Cancer (ref = not present)		0.78 (0.71–0.86)	<.0001	0.80 (0.71–0.90)	0.0002		
Aphasia (ref = not present)		1.50 (1.34–1.68)	<.0001	1.22 (1.05–1.43)	0.01	1.89 (1.60–2.23)	<.0001
Antibiotic resistant infection (ref = not present)		1.43 (1.28–1.59)	<.0001	1.55 (1.36–1.76)	<.0001		
HIV infection (ref = not present)		2.34 (1.48–3.69)	0.0003	2.95 (1.87–4.66)	<.0001		
Pneumonia (ref = not present)		0.82 (0.72–0.94)	0.004	0.73 (0.61–0.86)	0.0003		
Unsteady gait (ref = not present)		0.74 (0.69–0.79)	<.0001	0.70 (0.65–0.76)	<.0001	0.79 (0.71–0.88)	<.0001
Therapies							
Feeding tube (ref = no)		1.56 (1.37–1.78)	<.0001	1.42 (1.20–1.68)	<.0001	1.87 (1.53–2.29)	<.0001
Dialysis (ref = no)		1.54 (1.24–1.90)	<.0001	1.57 (1.23–1.99)	0.0003		
Psychological therapy [3] (ref = no)		1.64 (1.52–1.78)	<.0001	1.47 (1.33–1.62)	<.0001	1.70 (1.49–1.95)	<.0001
Tracheostomy, ventilator or respirator (ref = no)		1.89 (1.50–2.39)	<.0001	2.11 (1.63–2.73)	<.0001	2.07 (1.35–3.18)	0.0008
Discharge potential							
Desire to return to community (ref = no)		0.42 (0.39–0.45)	<.0001	0.44 (0.40–0.49)	<.0001	0.58 (0.51–0.65)	<.0001
Support person positive towards discharge (ref = no)		0.63 (0.58–0.68)	<.0001	0.54 (0.48–0.61)	<.0001	0.77 (0.68–0.87)	<.0001
C Statistic		0.76		0.77		0.74	

[1] Changes in Health, End-Stage Disease, Signs, and Symptoms Scale

[2] In last 90 days

[3] On 1+ days in last 7 days

Comparing patients discharged to community settings to those discharged to LTC facilities reveals some differences in the long-stay patient status risk factors. Female sex was a statistically significant predictor of long-stay designation among community discharges; however, this effect was not significant among LTC discharges. Community discharges with moderate or worse cognitive impairment (CPS 3+) had greater odds of being long-stay patients. Conversely, LTC discharges at the same level of cognitive impairment had lower odds of being long-stay patients. Among patients that were discharged to LTC facilities, aggressive behaviour severity, measured using the Aggressive Behaviour Scale, was a significant predictor of long-stay patient status. Aggressive behaviour was not a significant effect in the overall and community discharge models. Patients admitted to an LTC facility within the previous five years were less likely to become long-stay patients in the LTC discharge model, but this effect was not significant in the community discharge model. Quadriplegia was not a predictor of long-stay designation among community discharges, but it was a strong predictor among patients discharged to LTC. The opposite was true for cancer, antibiotic resistant infection, HIV infection, and pneumonia, which were predictors of long-stay designation among community discharges, but not among LTC discharges.

## Discussion

Numerous clinical characteristics are associated with time to discharge from Ontario CCC facilities. This information should be used to guide care and discharge planning for patients in CCC facilities, and those receiving similar levels of care in other health systems, such as skilled nursing and inpatient rehabilitation facilities. Previous research has identified that discharge delays from acute care hospitals are primarily attributable to nursing home and long-term home care admission [15]. This information has limited utility for post-acute patients who are more likely to have a partner that is unable to provide home help, be unable to self-manage medications, and be dependent for bathing and other transfers [33], thereby reducing the likelihood of discharge without continuing care. In Ontario CCC facilities, only 13% of patients are discharged to the community without home care [4]. The present study identifies patient-level factors associated with prolonged length of stay that are specific to patient care pathways commonly followed by post-acute care patients. The findings from this study may provide information to care planners and policy makers working to improve patient flow at facility and system levels.

A number of common factors that are predictive of prolonged length of stay were identified for both discharge destinations; however, differences were noted in both in the magnitude and direction of these effects. For example, while increased functional impairment was predictive of long-stay designation for both discharge destinations, the magnitude of the effect was much higher among community discharges This may have been due to barriers in the organization and implementation of community supports and services for patients with greater dependency for the completion of activities of daily living [15]. Though moderate to severe cognitive impairment was associated with greater odds of long-stay designation for patients discharged to the community, it was protective against long-stay designation among LTC facility discharges. Given that most individuals in Ontario LTC facilities present with cognitive impairment [6], it is unlikely to act as a barrier to discharge for LTC facility-bound individuals. Instead, factors that may present more complex situations for care (e.g., aggressive behaviour) influence long-stay designation among discharges to LTC. Aggressive behaviour is associated with cognitive impairment [26] and is a source of unit disruption and cause of staff burnout [34]. Therefore, finding a good residential fit for persons with aggressive behaviour may increase time to placement.

The identification of factors associated with long stays using standardized assessment information available upon admission to care creates an opportunity for early discharge planning and intervention for older adults. Previous studies have found that delayed discharges are often attributable to family negotiations [35] and requests for extension of hospital-stay by family [36]. The findings from this study are critical for targeting patients that would benefit from earlier and more intensive discharge planning, an advantage given that coordination among informal caregivers, community care services, and institutional care providers may require significant lead time to prevent delayed discharge. There are few decision-support tools available to assist discharge planners in identifying patients at risk of delayed discharge in post-acute care. The Blaylock Risk Assessment Screening Score is an index-based tool designed to identify hospital patients at risk of prolonged hospital stay and difficulties after discharge [37]. As it was developed for use in an acute care setting, when applied in a post-acute rehabilitation setting it demonstrates poor reliability, limiting its utility for identifying patients likely to experience post-discharge difficulties [38]. The interRAI suite of assessment tools generate a series of Clinical Assessment Protocols (CAPs) based on assessment inputs to aid clinicians in implementing care plans in response to areas of identified clinical need [19]. At this time, a CAP that is specific to post-acute care transitions does not exit; however, the prolonged length of stay risk factors that were identified in this study may serve as the foundation for a decision-support algorithm of this type.

This study holds a number of implications for health system administrators. For those that are focused on quality improvement, the results of this study may serve as the foundation for risk-adjusted discharge planning benchmarks, allowing for fair facility comparisons based on discharge planning efficacy after accounting for risk factors associated with prolonged length of stay. These risk-adjusted performance measures may be used for program evaluation and, and through public reporting, could incentivize discharge planning quality improvement across the health system. In terms of health system planning, the results of this study may be useful for optimizing patient flow along the continuum of care. Patients aged 65 years and older had lower odds of long-stay designation. Similarly, New et al. [35] found that patients aged 50 years and younger had greater odds of experiencing a discharge barrier from a rehabilitation facility. After accounting for clinical status, younger patients are likely at greater odds of long-stay designation as a result of a lack of long-term facility-based care settings that are oriented towards caring for younger individuals. Although advanced age is not a criterion for admission to an institutional care setting in Ontario, few LTC residents are younger than age 65 [6]. We also found that patients requiring a feeding tube, dialysis, tracheostomy care, or a ventilator are at increased odds of long-stay designation. Though there are few patients in CCC facilities that require these therapies, capacity to provide accommodations outside of hospital-based care may be limited or require extensive coordination with community care providers. For example, the discharge barriers most frequently experienced by community-bound mechanically ventilated patients relate to public funding arrangements and a shortage of paid caregivers [39]. These findings support the implementation of alternative models of community and residential care that meet the preferences and needs of both younger adult patients and those that require technically advanced medical treatments that can be provided outside of hospital settings. Examples include intensive home health care [40, 41] and small-scale shared supported accommodations [42].

Recently, much of the discourse around patient flow in Ontario is focused on reducing both the number of ALC patients and the proportion of total patient days that ALC patients consume in Ontario hospitals. Unfortunately, ALC patient information is not available in the CCRS data repository. This study focused on identifying those patients that consume the greatest number of patient days and who were believed to be most likely to experience discharge barriers from CCC facilities. This approach was employed because it is reasonable that a large proportion of these long-stay patients are also ALC patients. While others have used resource intensity measures to identify “low-care” cases, without data linkages to ALC data sources [43, 44], identifying patients that would best be cared for in a more appropriate setting is challenging. Future studies should focus on identifying the clinical characteristics and discharge barriers associated with ALC patient status as it is a direct indicator of delayed hospital discharge.

Regional market factors (e.g., population density and proximity to community-based supports) can have an impact on rates of discharge and adjacent care setting admission rates [45]. This study did not consider regional variation in length of stay. Though stratifying these analyses by each of Ontario’s fourteen Local Health Integration Networks (i.e., regional health authorities) is feasible, differentiating urban from rural facilities may be sufficient to observe the effect of facility location and proximity to other services on length of stay. Future studies may seek to account for group effects through multilevel modelling approaches. Similarly, this study was not able to account for intrinsic facility factors such as the number of patient beds, patient bed types, program offerings, occupancy and staffing that are not collected using the MDS 2.0 assessment, but are known to affect patient transitions [45].

This study used a large sample of administrative health records that provide near census-level representation of CCC admissions over a twelve-year time period. With increased power attributable to large sample size, many small group differences were statistically significant. Therefore, when interpreting the findings from this study, emphasis should be placed on the magnitude of the group differences and odds ratios. The risk factors for prolonged length of stay in this study were identified using only the MDS 2.0 assessment that is completed at admission to CCC. While this allows discharge planners to identify probable long-stay patients early in the episode of care, the results of this study do not account for changes in patient health status that may contribute to long-stay designation. Additionally, given that the sample only included patients that were discharged to residential long-term care and community settings, it does not account for long-stay patients that were not discharged, died in CCC, or transitioned to other service settings including acute, rehabilitation and psychiatric care facilities.

## Conclusion

The results of this research indicate that several patient attributes and process variables serve as both predictors of length of stay and discharge barriers from CCC facilities. Although the manner in which these variables operate is complex and the length of time associated with long-stay designation differs by discharge destination, a set of common clinical characteristics are associated with long-stay designation in CCC facilities. This research may serve as a foundation for health system planning and the development of discharge planning decision-support algorithms and facility bench marking tools that may reduce the number of protracted discharged in post-acute care settings.
